# Identification of Prognostic Markers of N6-Methylandenosine-Related Noncoding RNAs in Non-Small-Cell Lung Cancer

**DOI:** 10.1155/2022/3657349

**Published:** 2022-04-01

**Authors:** Zexin Zhang, Jing Li, Ke Lu, Wenfeng Wu, Ziyi Huang, Chi Zhang, Wei Guo, Jiayin Li, Lizhu Lin

**Affiliations:** ^1^The First Clinical Medical College of Guangzhou University of Traditional Chinese Medicine, Guangzhou, China; ^2^The First Affiliated Hospital of Hunan University of Traditional Chinese Medicine, Changsha, China; ^3^The Second Clinical Medical College of Guangzhou University of Traditional Chinese Medicine, Guangzhou, China; ^4^The First Affiliated Hospital of Guangzhou University of Chinese Medicine, Guangzhou, China; ^5^Cancer Project Team of China Center for Evidence Based Traditional Chinese Medicine, Guangzhou, China

## Abstract

**Background:**

Non-small-cell lung cancer (NSCLC) is a major type of lung carcinoma that threatens the health and life of humans worldwide. We aimed to establish an n6-methyladenosine (m6A)-relevant ncRNA model to effectively evaluate the outcome of patients.

**Methods:**

m6A-Related ncRNAs (lncRNA/miRNA) were acquired from the UCSC Xena database. Pearson's correlation analysis among 21 m6A regulatory factors and ncRNAs were implemented to explore m6A-relevant ncRNAs. Weighted gene co-expression network analysis (WGCNA) identified hub modules of gene associated with prognosis of NSCLC patients. Univariate Cox regression analysis identified 80 m6A-related ncRNAs. Least absolute shrinkage and selector operation (LASSO) filtered out redundant factors and established a risk score model (m6A-NSCLC) in the TCGA training data set. Validation of prognostic ability was performed using testing data sets from the TCGA database. We also conducted a correlation analysis among the risk score and different clinical traits. Both univariate and multivariate Cox analyses were combined to verify prognostic factors which have independent value, and a nomogram on the basis of m6A-NSCLC risk scores and clinical traits was constructed to assess the prognosis of patients. In addition, we screened differentially expressed genes (DEGs) based on different risk scores and performed enrichment analysis. Finally, 21 m6A regulators were detected to be differentially expressed between two risk groups.

**Results:**

An m6A-NSCLC risk model with 18 ncRNAs was constructed. By comparison with low-risk patients, high-risk score patients had poor prognosis. The distribution of risk score in the tumor size and extent (*T*), number of near lymph nodes (*N*), clinical stage, sex, and tumor types was significantly different. The risk score could act as an independent prognostic factor with the nomogram assessing overall survival in NSCLC. DEGs inherent to cell movement and immune regulation were involved in NSCLC development. Furthermore, 18 of 21 m6A regulators were differentially expressed, implying their correlation to survival prognosis.

**Conclusion:**

The m6A-NSCLC could be effectively utilized for evaluation of prognosis of patients.

## 1. Introduction

Lung cancer is one of the most prevalent malignancies threatening the health and life of humans in the world. Studies have demonstrated that non-small-cell lung cancer (NSCLC) is the major pathological type of lung cancer, with lung adenocarcinoma (LUAD) and lung squamous cell carcinoma (LUSC) accounting for approximately 85% of NSCLC cases [1]. The prognosis for patients with LUAD is very poor, with a 5-year survival rate of less than 15% [2]. In addition, the incidence rate of LUSC, which has a poor response to therapy, high recurrence rates, and poor prognosis, is second only to that of LUAD [3, 4]. At present, surgery remains the main approach for the treatment of NSCLC. However, adjuvant treatments mainly involve chemotherapy, targeted therapy, and immunotherapy [5, 6]. Despite the apparent shrinkage in tumor volume and size and prolonging patient survival, these treatments have been reported to cause unavoidable deleterious side-effects, such as nausea, bone marrow suppression, and vomiting, which seriously impact the quality of life in NSCLC patients [7–9].

To date, studies on n6-methyladenosine (m6A) are ongoing. In particular, m6A has been proved to play a pivotal role in the splicing, export, translation, and stability of RNAs [10]. More importantly, the m6A modification which is known to be regulated by various factors, including “readers” (signal transducers), “writers” (methyltransferases), and “erasers” (demethylases), was shown to be highly relevant to the onset and progression of NSCLC [11]. For example, Jin et al. revealed that methyltransferase like 3 (METTL3) facilitated expression and activity of Yes-associated protein (YAP), inducing drug resistance and metastasis of NSCLC [12], whereas ALKBH5 repressed the expression of YT521-B homology domain-containing family (YTHDF)-mediated YAP and inhibited the activity of miR-107/LATS2-mediated YAP, thus suppressing the growth and metastasis of lung tumors [13]. Shi et al. experimentally confirmed that lacking of YTHDF1 was able to restrain NSCLC cells proliferating and xenograft tumors formating [14].

Furthermore, noncoding RNAs (ncRNA: lncRNA/miRNA) have been demonstrated to play pivotal roles in the progression of a lot of cancers, and multiple studies have demonstrated that dysregulation of ncRNAs could affect the progression of NSCLC [15]. In the cytoplasm, lncRNAs are known to influence the stability and translated regulation of mRNA mainly through the adsorption of miRNAs [16]. Moreover, lncRNAs have been shown to bind not only their own miRNAs, but also miRNA binding sites on their target mRNAs, hence further regulating the activity of their target miRNAs [17]. In contrast, miRNAs are known to target lncRNAs to alter their stability, thereby mediating their abundance, and affecting different cellular processes [18]. Therefore, the attention for lncRNAs and miRNAs has increased in tumor studies. For instance, knockdown of the DLEU2 lncRNA was found to inhibit the expression of SOX9 and the onset and development of NSCLC through up-regulation of miR-30c-5p [19]. High levels of SNHG1 were shown to act as a tumorigenic lncRNA, accelerating the occurrence of NSCLC tumors by curbing miR-101-3p and actuating the Wnt/*β*-catenin pathway [20]. Moreover, bladder-cancer-associated transcript 1 (BLACAT1) was reported to stimulate the expression of ATG7 by miR-17 and promote autophagy and drug resistance in NSCLC cancer cells [21]. Although the interaction between m6A modifications and the dysregulation of ncRNAs remains unknown, few researches have attempted to delve the mechanism of m6A-related ncRNAs in NSCLC. Therefore, understanding the mechanism by which m6A-relevant ncRNAs in the progression of NSCLC is able to help identify biomarkers and might also serve as a useful therapeutic strategy.

In this study, we identified m6A-relevant ncRNAs for the prognosis of NSCLC based on TCGA training (*n* = 705) and TCGA testing (*n* = 301) data sets, using bioinformatics and statistical analyses. Moreover, we established an m6A-relevant ncRNA risk score model (m6A-NSCLC) on the basis of the ability of 18 m6A-related ncRNAs to predict the survival status of patients with NSCLC and identified independent prognostic factors and constructed a nomogram for predicting overall survival (OS) in NSCLC patients. Furthermore, differentially expressed genes (DEGs) involved in enrichment analysis were analyzed to explore the connections between cell movement and immune regulation and the development of carcinoma. Finally, a total of 21 m6A regulatory factors were investigated to determine whether differences between the two risk groups are significant.

## 2. Materials and Methods

### 2.1. Data Downloading and Processing from UCSC Xena Database

**Figure 1:**
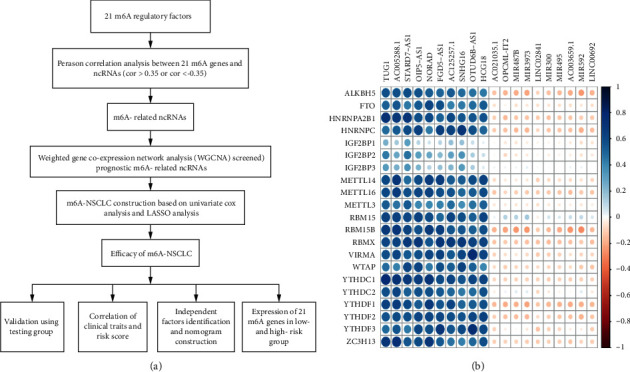
Technology roadmap and m6A-related ncRNAs: (a) technical roadmap of the study and (b) heatmap of the correlations between 21 m6A regulatory factors and the ncRNAs of NSCLC.

The expression, phenotype, and survival data of NSCLC were obtained from the UCSC Xena database (https://xenabrowser.net/), while information on ncRNAs was acquired from the GTF file (Homosapiens.GRCh38.99.gtf.gz) downloaded from the Ensemble database (http://www.ensembl.org/info/data/ftp/index.html). TCGA-NSCLC included both LUAD and LUSC cases. Eventually, TCGA data sets corresponding to 1006 patients with NSCLC were obtained from the UCSC Xena database. The technology roadmap is shown in Figure 1(a). The Cart package in *R* was applied to divide these patients into training (705) and testing groups (301), as shown in Table 1. The expression matrix of 21 m6A regulatory factors was extracted from the training group. Pearson's correlation analysis with the standard of correlation coefficient >0.35, and *p* < 0.05, identified m6-related ncRNAs. Finally, these m6A-related ncRNAs were subjected to WGCNA.

### 2.2. Identification of Prognosis Modules of m6A-Related ncRNAs Based on Weighted Gene Co-Expression Network Analysis (WGCNA)

The gene modules related to the survival status of NSCLC were distinguished by WGCNA package in *R*. WGCNA is a method that was used to analyse gene expression patterns of multiple samples. Genes with similar functions are clustered together, and the relationships among different modules and clinical traits or phenotypes can be analyzed. WGCNA is applied in the analysis of diseases, traits, and gene associations widely. The pickSoftThreshold function was applied to select an optimum soft power ratio *β* to establish a scale-free network. Using the formula AIJ = | SIJ | *β*, this was transformed into a topological overlap matrix (TOM) and its related dissimilarity degree (1-TOM) (AIJ: adjacency matrix between gene I and gene J, SIJ: similarity matrix obtained by Pearson's correlation of all gene pairs, *β*: soft power value). The gene modules that correlated significantly with NSCLC clinical traits were retained and displayed in different colors. We then chose the most significant gene modules of the prognosis of patients for the subsequent construction of the risk model.

### 2.3. Construction and Evaluation of the Risk Model in m6A-Related ncRNAs of NSCLC

Gene modules significantly relevant to the prognosis outcome of patients with NSCLC were further analyzed using univariate Cox regression analysis with a standard *p* value < 0.05 through the survminer and survival package of *R*. To establish a robust and accurate risk model, we subsequently carried out a least absolute shrinkage and selector operation (LASSO) analysis to filter out redundant factors through the glmmet package in *R*. The lambda value was screened through cross-validation, and the model was constructed using the lambda.min value. The gene expression matrix was extracted from this model, and each sample' risk score was calculated by the following formula:

(1) $$\begin{array}{c} {\text{RScore}}_{i}=\sum_{j=1}^{n}\exp_{ji}\times \beta_{j}, \end{array}$$

where exp represents the expression of the corresponding genes, *β* is the regression coefficient (coef), and *i* stands for sample, whereas *j* stands for gene. Summation of multiple samples by genes led to the final risk score. According to the risk score, we divided the model into two risk groups based on the median. The calculated area under the curve (AUC) of the obtained ROC curve was applied to evaluate our risk model. The testing group confirmed the prediction ability of our model.

### 2.4. Correlation Analysis between m6A-NSCLC and Clinical Factors

In order to explore the clinical indicators related to the m6A-NSCLC risk score, we combined the TNM stage, clinical stage, age, sex, and tumor type of NSCLC, which are broadly applied in the evaluation of clinical prognosis and are beneficial for guiding the treatment of patients.

### 2.5. Validation of Independent Prognostic Factors and Construction of Nomogram Based on Univariate and Multiple Cox Analysis

To verify whether risk score was a prognostic factor that has independent value or combined with other prognostic factors, including age, gender, pathologic *M*, pathologic *N*, and pathologic *T*, we performed univariate and multiple Cox analyses. Clinical indicators with significant differences in both univariate and multivariate Cox analyses were considered independent prognostic factors. Furthermore, we established a nomogram formulating scoring standards on the basis of the regression coefficients of all independent variables and providing a score for each value level of independent variables. Using this nomogram, we calculated a total score for patients with NSCLC, and transformed the scores to the odds of a resulting function to calculate the probability of the outcome time of each patient. The drawing of the nomogram was based on the ms and survival packages of *R*. We first constructed the Cox proportional risk regression model using the cph function, then calculated the survival probability using the survival function, and finally constructed the objects using the nomogram function. Calibration and multivariate ROC predictive curves were also drawn to evaluate their reliability.

### 2.6. Differentially Expressed Genes and Function Enrichment Analysis between High- and Low-Risk Groups

Using the m6A-NSCLC risk score, we set the criteria of |logFC| > 1 and FDR < 0.05 for differential expression analysis to distinguish genes with specificity in the high- and low-risk groups using the edgeR package in *R*. We investigated the biological processes of these DEGs and also carried out functional enrichment analysis through Metascape online database (http://metascape.org/gp/index.html#/main/step1) to dig out the mechanics of their functions.

### 2.7. Expression Analysis of 21 m6A Regulatory Factors in High- and Low-Risk Groups

The expression matrix of m6A regulatory factors was extracted, and their differential expression in high- and low-risk groups was calculated. We used the Wilcox test to calculate the level of significant differences. Any differences identified in these m6A regulatory factors between risk groups might be related to the prognosis of NSCLC.

## 3. Results

### 3.1. Screening of m6A-Related Non-small-Cell Lung Cancer ncRNAs

We downloaded gene expression, phenotype, and survival data of NSCLC tumors from the UCSC Xena database. Following the integration of expression and survival information, the final NSCLC data set encompassed 1006 samples, which were further divided into training (701) and testing (305) groups. We also extracted information on 14,899 ncRNAs from the GTF profile and then integrated them into the expression matrix. By calculating the correlation between 21 m6A regulatory factors and ncRNAs using the criteria of correlation coefficients > 0.35 and *p* value < 0.05, we finally screened 4208 m6A-related ncRNAs. Accordingly, m6A-related ncRNAs with positive and negative correlations ranking in the top 10 were drawn in a heatmap (Figure 1(b)).

### 3.2. Identification of Prognosis Modules of m6A-Related ncRNAs Based on Weighted Gene Co-Expression Network Analysis (WGCNA)

**Figure 2:**
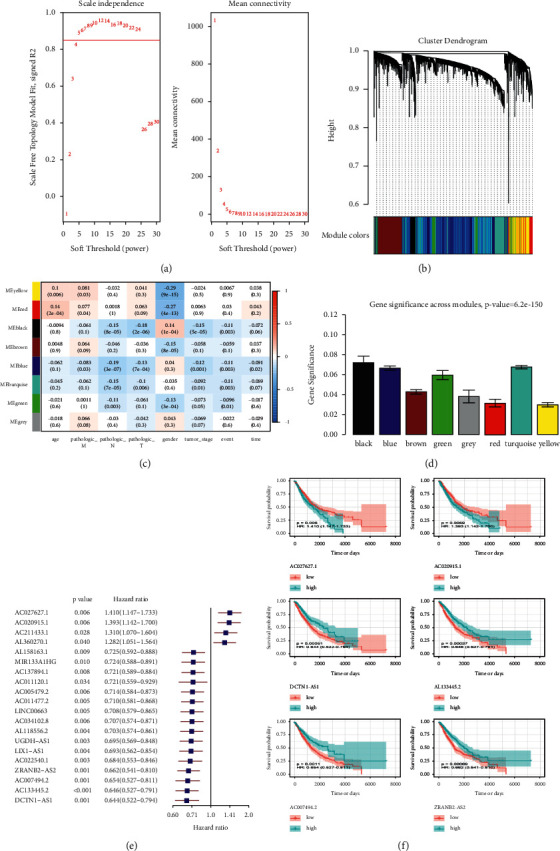
Identification of prognostic gene modules of m6A-relevant ncRNAs based on weighted gene co-expression network analysis (WGCNA): (a) screening of the best soft-thresholding, (b) cluster dendrogram of gene modules, (c) correlation analysis among clinical traits and gene modules, (d) gene significance across modules, (e) prognosis-related ncRNAs screened by univariate Cox regression analysis, and (f) drawn of KM survival curve of the 6 ncRNAs.

We then subjected the obtained 4208 ncRNAs and clinical data of the training group (705) to WGCNA analysis. We used the *R*^2^ > 0.85 as the standard to screen soft threshold 5 (Figure 2(a)) and then constructed the network to obtain eight (8) modules (Figure 2(b)). In the correlation analysis of the 8 gene modules, we included prognostic factors, such as age, sex, TNM stage, clinical stage, event, and time. Our results showed that the black and blue modules were the most significant modules relevant to the outcome of NSCLC patients with a *p* < 0.003 and correlation coefficient of 0.11 (Figure 2(c)). We then calculated the correlation matrix between ncRNAs of the eight modules and the outcome of patients to obtain gene significance (GS) (Figure 2(d)). We found that the black and blue modules showed the highest score, ranking first and second, respectively. Consequently, we used these two gene modules with 1139 ncRNAs for the construction of the risk model.

### 3.3. Construction of Risk Model of m6A-Related ncRNA

**Figure 3:**
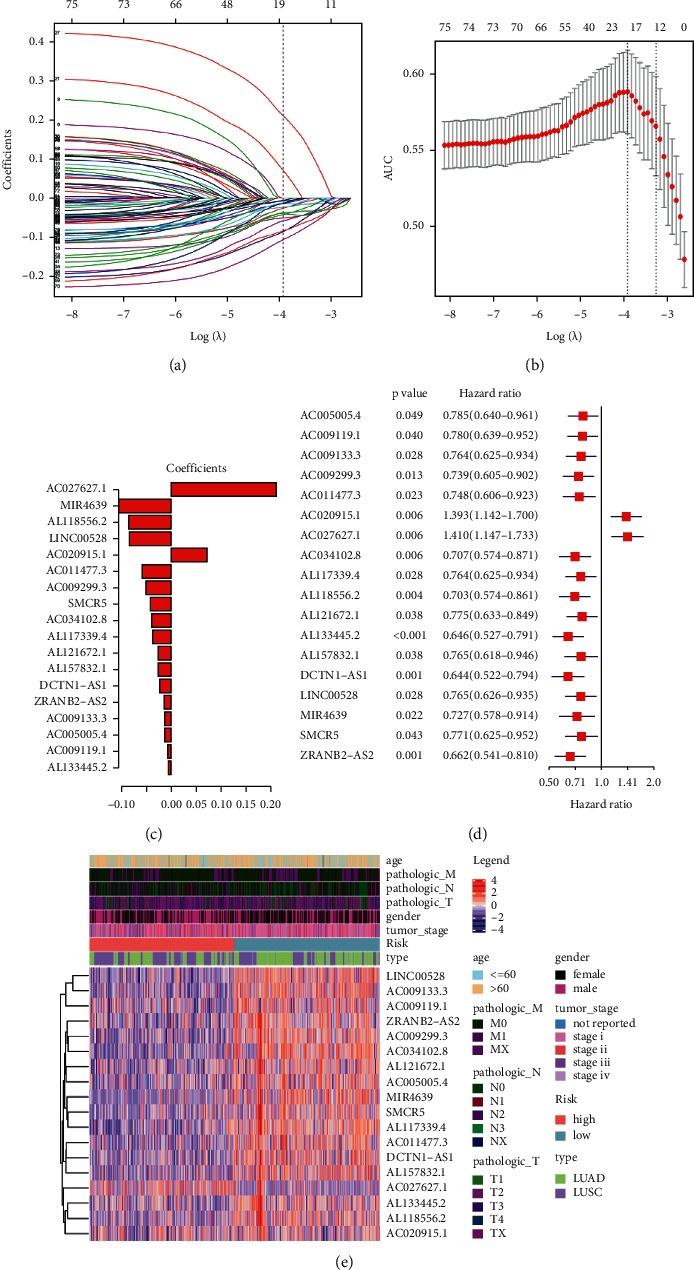
Construction of the risk model in m6A-related ncRNAs of NSCLC: (a) Lambda.min through cross-validation filtered out redundant factors, (b) weighted gene co-expression network analysis (WGCNA)-confirmed number of ncRNAs, (c) coefficients of 18 m6a-related ncRNAs, (d) forest plot of m6A-NSCLC, and (e) heatmap of the associations between the expression levels of the nine m6A-related ncRNAs and clinical traits in the TCGA training data sets.

Using univariate Cox analysis based on the 1139 ncRNAs, we identified 80 m6a-related ncRNAs with prognostic ability (Figure 2(e)). These ncRNAs with *p* < 0.05 ranking in the top 4 in the low-risk score and top 2 in the high-risk score were drawn in a Kaplan–Meier (KM) survival curve (Figure 2(f)). Our results revealed a greater drop in the survival curve of the AC027627.1 and AC020915.1 in the high-risk group, indicating a lower survival rate with time. In contrast, other ncRNAs, such as DCTN1-AS1, AL133445.2, AC0077494-2, and ZRANBS-AS2, exhibited a greater drop in the survival curves in the low-risk group. Interestingly, these ncRNAs exhibited the same trend whether in univariate Cox or KM survival analysis (hazard ratio <1 or >1.95% confidence intervals), suggesting a stable predictive effect. Subsequent LASSO analysis filtered out redundant factors (Figures 3(a) and 3(b)). Using lambda.min through cross-validation, we screened 18 m6a-related ncRNAs and incorporated them in a risk model (Figures 3(c) and 3(d)). The optimized model was as follow: Risk score = MIR4639 × (−0.1065) + AL118556.2 × (−0.0860) + LINC00528 × (−0.0840) + AC011477.3 × (−0.0586) + AC009299.3 × (−0.0510) + SMCR5 × (−0.0423) + AC034102.8 × (−0.0399) + AL117339.4 × (−0.0378) + AL121672.1 × (−0.0261) + AL157832.1 × (−0.0261) + (DCTN−AS1) × (−0.0233) + (ZRANB2−AS2) × (−0.0138) + AC009133.3 × (−0.0134) + AC005005.4 × (−0.0134) + AC009119.1 × (−0.0074) + AL133445.2 × (−0.0061) + AC020915.1 × 0.0720 + AC027627.1 × 0.2116. We calculated the risk score of all samples and divided them into high- and low-risk groups based on the median value. The distribution of clinical traits between two risk groups showed in Figure 3(e). We thus obtained 353 high- and 352 low-risk samples for subsequent analysis.

### 3.4. Predictive Efficacy Analysis of Prognostic Model

**Figure 4:**
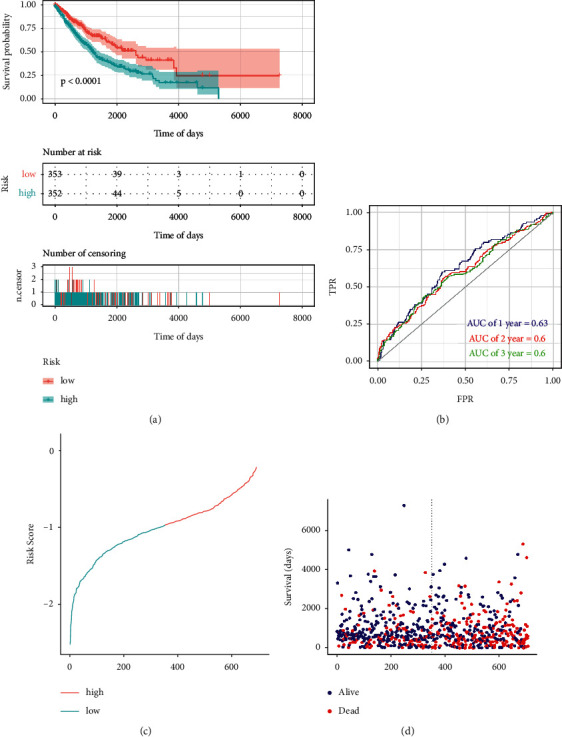
Efficacy evaluation of training group: (a) KM survival analysis between high- and low-risk group, (b) area under ROC curve of 1, 2, and 3 years, (c) change of risk score over time, and (d) change of survival days and survival status over time.

**Figure 5:**
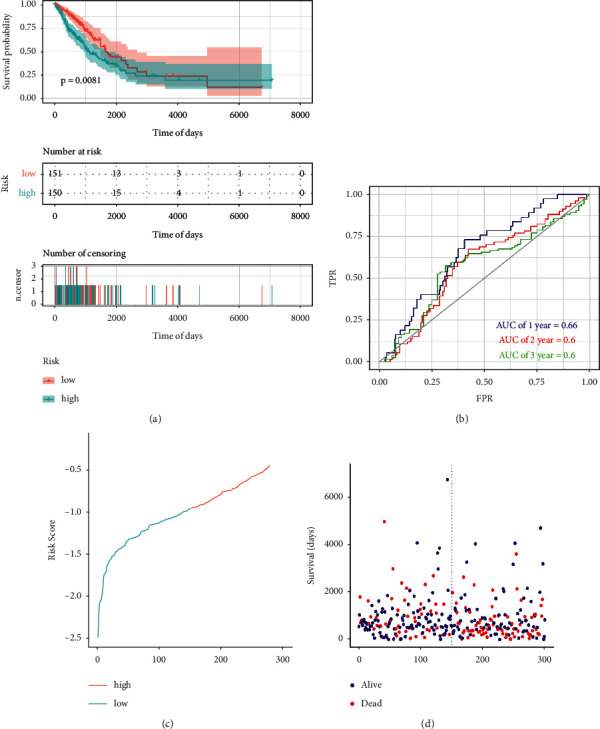
Efficacy evaluation of testing group: (a) KM survival analysis between high- and low-risk group, (b) area under ROC curve of 1, 2, and 3 years, (c) change of risk score over time, and (d) change of survival days and survival status over time.

To validate the predictive efficacy of our risk model, we used KM analysis to calculate the survival difference between the two risk groups. We found that the low-risk group exhibited better survival compared with the high-risk group over time (*p* < 0.0001) (Figure 4(a)) and the mortality of patients increased with the increase in the risk score (Figures 4(c) and 4(d)). We then drew the ROC curve of 1, 2, and 3 years and observed that the AUC was 0.63, 0.6, and 0.6, respectively, further indicating the good prognostic prediction efficacy of the m6A-NSCLC risk model (Figure 4(b)). We used the testing group to verify the reliability of the m6A-NSCLC model using the same coefficient as that in the training group and noticed significant differences between the high- and low-risk groups (*p* < 0.0081) (Figure 5(a)). Simultaneously, calculation of the AUC of the ROC curve showed that the AUCs of 1, 2, and 3 years were 0.66, 0.6, and 0.6, respectively, effectively verifying the efficacy of our model (Figure 5(b)). Similarly, the mortality of patients increased with the increase in the risk score (Figures 5(c) and 5(d)).

### 3.5. Relationship of m6A-NSCLC with Different Clinical Traits

**Figure 6:**
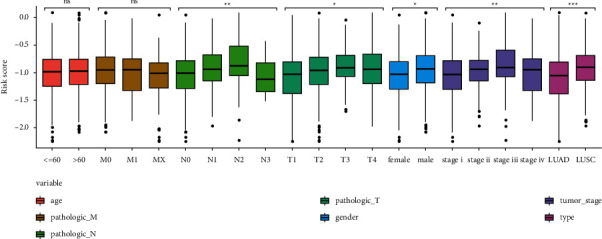
Distribution of risk score in different clinical traits. ^*∗*^<0.05, ^*∗∗*^<0.01, ^*∗∗∗*^<0.001.

We observed that tumor infiltration, lymph node, and metastasis were the three main clinical traits correlated with the prognosis of patients, whereas age and sex were found to exhibit inconsistent results regarding the occurrence and development of carcinomas. Here, the risk score of all samples was calculated according to age, sex, TNM stage, clinical stage, and tumor types (LUAD and LUSC). We found that the risk scores for sex, TN stage, clinical stage, and tumor type were significantly different, suggesting that the consistency of these clinical traits with our risk score; LUSC was observed to exhibit a higher risk scores than LUAD and poor prognosis compared with LUAD (Figure 6).

### 3.6. Risk Score Was an Independent Prognostic Factor through Univariate and Multiple Cox Analyses

**Figure 7:**
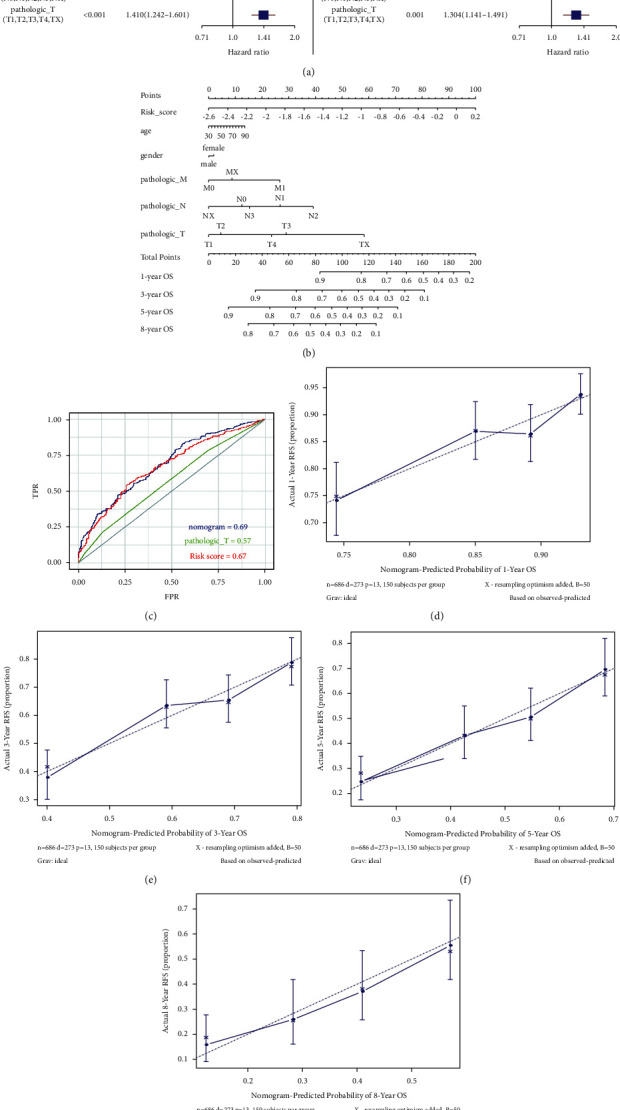
Validation of independent prognostic factors and construction of nomogram based on univariate and multiple Cox analysis: (a) univariate Cox regression and multivariate Cox regression analysis, (b) nomogram of different clinical traits of 1, 3, 5, and 8 years, (c) area under ROC curve of nomogram, risk score, and pathologic *T*, and (d-g) calibration curve of nomogram of 1, 3, 5, and 8 years.

In order to validate the predictive efficacy of the risk score, we conducted univariate and multiple Cox regression analyses incorporating age, sex, and TNM stage. Our results showed that the risk score had the highest hazard ratio (HR) and *p* value < 0.001, thus exhibiting the best efficacy. Other factors, such as T stage and N stage were found to be second to risk score with HR > 1, exhibiting a steady ability. In contrast, the 95% confidence interval across 1 and *p* value > 0.05 of the remaining factors suggested their poor ability for prognosis (Figure 7(a)). We subsequently used the aforementioned factors to construct a nomogram (Figure 7(b)). As the nomogram showed, the contribution of the risk score was the highest, with the AUC of the nomogram and risk score being 0.69 and 0.67, indicating their powerful prognostic ability (Figure 7(c)). Moreover, the simulation curve of the nomogram is almost distributed around the diagonal, which indicates that the model has a good fitting degree (Figures 7(d)–7(g)).

### 3.7. Differentially Expressed Genes in Functional Enrichment Analysis

**Figure 8:**
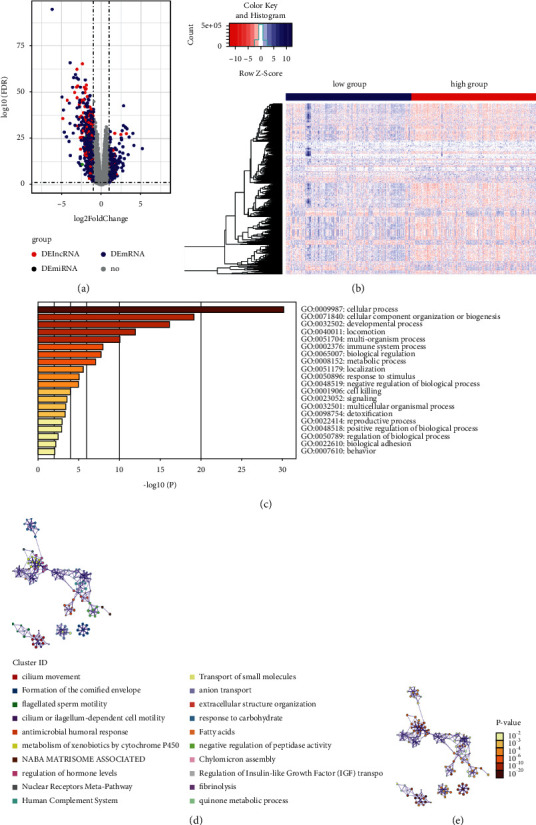
Differentially expressed genes and function enrichment analysis between high- and low-risk groups: (a) volcano map identified differential expressed genes between high- and low-risk group, (b) heat map identified differential expressed genes between high- and low-risk group, (c) Bar plot of enrichment analysis showed biological processes with *p* value ranking top 20, (d) items of network diagram of interacting biological processes, and (e)*p* value of interacting biological processes.

According to the criteria of |FDR| > 1 and *p* value < 0.05, we identified 1465 DEGs, which included 826 mRNAs, 6 miRNAs, and 426 lncRNAs (Figures 8(a) and 8(b)). We observed that more genes were down-regulated in the high-risk group than the low-risk group, indicating a close correlation among high-risk samples and the downregulating genes. We then used the Metascape online analysis tool for functional enrichment analysis of 826 different mRNAs. We found that cellular processes, cellular component organization or biogenesis, developmental processes, locomotion and immune system, and process response to stimulus were the main mechanisms, suggesting the importance of genes involved in cell movement and immune regulation (Figures 8(c)–8(e)).

### 3.8. Significant Differential Expression of m6A Regulatory Factors in Risk Groups

**Figure 9:**
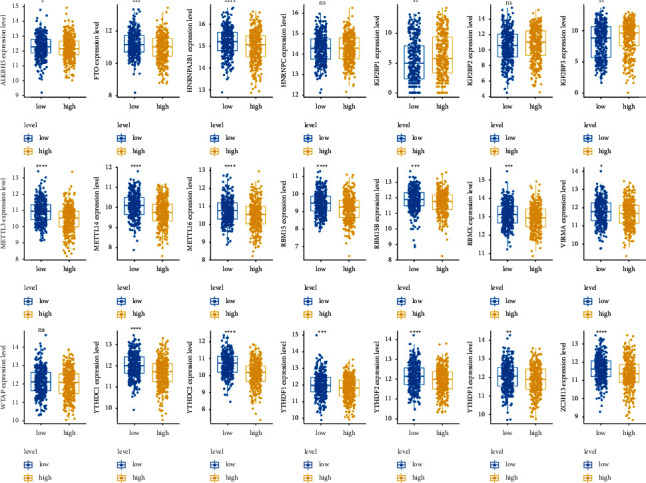
Expression level of 21 m6A regulatory factors in high- and low-risk group. ^*∗*^<0.05, ^*∗∗*^<0.01, ^*∗∗∗*^<0.001, and ^*∗∗∗∗*^<0.0001.

In order to determine whether the 21 m6A regulatory factors were consistent with our risk score, we also performed a different analysis, which showed that 18 of 21 m6A regulatory factors were differentially expressed between the high- and low-risk groups. This finding suggested that the level of expression of these 18 m6A regulatory factors might be relevant to the prognosis of patients with NSCLC (Figure 9).

## 4. Discussion

In this study, we established a prognostic model of m6A-related noncoding RNAs in 1006 patients with NSCLC from TCGA training and testing data sets. Among them, 80 m6A-related ncRNAs were confirmed to have prognostic value, and 18 of them were used in an m6A-NSCLC model to predict the survival status of patients with NSCLC. The performance of m6A-NSCLC was evaluated by dividing the NSCLC samples into high- and low-risk subgroups, using the median risk score as a cutoff. The log-rank test of KM survival analysis showed significant differences between the two subgroups, and the calculated area under the ROC curve demonstrated the reliability of the model. Investigation of the relationship of the m6A-NSCLC model with different clinical traits revealed that sex, TN stage, clinical stage, and tumor type were consistent with our risk score. Interestingly, compared with LUAD, LUSC had a higher risk score, implying a worse outcome of patients. Simultaneously, univariate and multivariate Cox regression analysis showed that risk score was an independent prognostic factor with the highest hazard ratio. Other independent prognostic factors encompassed *T* and *N* stage, which also showed a steady ability of prognosis. We observed that the area under the ROC curve of the risk score and nomogram were 0.67 and 0.69, further verifying their prognostic efficacy. Functional enrichment analysis revealed that DEGs were enriched in cell movement and immune regulation in NSCLC. Finally, analysis regarding the expression of m6A regulatory factors towards high- and low-risk groups implied that 18 of 21 m6A regulatory factors were possible relevant to the prognosis of NSCLC.

Indeed, a plethora of studies have demonstrated that m6A is the most abundant modification in mRNAs, [22] and its regulatory factors have been reported to play pivotal roles in the occurrence and development of tumors through various mechanisms, [23] thus providing more evidence for the early diagnosis and treatment of cancer [24]. However, the biological roles of ncRNAs (lncRNA/miRNA) in the onset and development of NSCLC, and the relationship between the expression of m6a-relevant ncRNAs and prognosis have not been fully clarified [25]. For instance, m6A was shown to modify specific ncRNAs to maintain the survival of malignant tumor cells by affecting the production/metabolism of RNAs. The alkylation repair homologous protein 5 (ALKBH 5) is a demethylase that can mediate the reversal of methylation. The combination of ALKBH 5 and NEAT 1 was reported to promote demethylation of the NEAT 1 lncRNA, giving a result to the metastasis and invasion of gastric cancer cells [26]. YTHDF 3, which belongs to the YTHDF family, is known to be a “reader” of m6A. In colorectal cancer tissues, the increase in the level of YTHDF 3 was reported to reduce the expression of the GAS 5 lncRNA, which is regulated by m6A [27] and related to colorectal cancer cells. MALAT 1 is known to bind to miR-204 by modifying and recognizing m6A, thereby up-regulating IGF2BP2 to facilitate the growth, migration, proliferation, and invasion of thyroid cancer cells [28]. Likewise, METTL 3-mediated m6A inhibited miR-647 through ZFAS 1 and promoted tumor growth and metastasis in cervical cancer [29].

The ncRNA study of m6A could be used as a pivotal factor for predicting the poor prognosis of tumours [30]. ncRNAs, as important regulators that target m6A modulators, [31] have significant carcinogenic relevance in various cancers and are expected to become new targets for tumor treatment [32]. In summary, we believe that m6a-related ncRNAs are strongly related to the prognosis of NSCLC, and are very likely to become new diagnostic biomarkers for NSCLC.

Using TCGA training data sets, we obtained 80 prognostic ncRNAs related to m6A from 705 patients with NSCLC, 18 of which were included in the m6A-NSCLC risk model. LINC00528 has been shown to be highly expressed in tumor tissues of patients with laryngeal squamous cell cancer and was related to the poor prognosis of patients [33]. ZRANB2-AS2 is a novel susceptibility gene for human anthropometric variation and has been demonstrated to be a clinical biomarker in glioblastoma that affects tumor development through mTOR signaling pathways [34]. miRNA4639 is a novel miRNA, and researches showed that plasma levels of hsa-miR-4639-5p in patients with Parkinson's disease were significantly up-regulated, making cells more susceptible to oxidative stress [35]. Current research on DCTN1-AS1 has mainly focused on the immune direction of Alzheimer's disease, with DCTN1 possibly physically connecting the EEF1A1-recognized cytoplasmic aggregates to the power protein motor and participating in the retrograde transport of the microtubule cargo [36]. A number of ncRNAs has been reported to be correlated with cancer or other diseases; nevertheless, there have been few studies on ncRNAs in NSCLC, and even more rare reports of m6A-related ncRNAs in NSCLC. Hence, we hope that our study will be beneficial to identify novel m6A-related ncRNAs, provide new strategy for the diagnosis of NSCLC, and elucidate the possible mechanics of its onset or development.

This study used The Cancer Genome Atlas (TCGA) data sets for analysis, including data collection, centralized data analysis, and validation in testing data sets. As we only carried data sets for validation, there were certain limitations to our study. Therefore, we acknowledge that more independent NSCLC data should be tested to further validate this hypothesis. Moreover, the molecular mechanism by which m6A-associated ncRNAs are involved in the development of NSCLC should be further explored both in vivo and in vitro. Consequently, our study provides a useful clue for further studies on NSCLC therapy.

## 5. Conclusion

In summary, we provided a strategy for evaluating the prognosis of patients with NSCLC utilizing an m6A-NSCLC model, which was an independent predictor relevant to OS in NSCLC.

## Figures and Tables

**Table 1:** Clinical information of 1006 NSCLC patients.

	train_data	test_data
Gender		
Male	430	172
Female	275	129

Age (years)		
Mean	66	67
Median	67	68

Tumor_stage		
Stage I	364	157
Stage II	187	91
Stage III	120	43
Stage IV	24	8
Not reported	10	2

Pathologic_M		
M0	517	230
M1	23	8
MX	158	61
NA	7	2

Pathologic_N		
N0	456	195
N1	151	70
N2	83	26
N3	4	3
NX	11	6
NA	0	1

Pathologic_T		
T1	191	94
T2	401	160
T3	78	38
T4	32	9
TX	3	0

## Data Availability

The data of this study were acquired from UCSC Xena and TCGA databases.
